# Interaction between mitochondrial TSPO and inflammatory responses in aging mouse hippocampus

**DOI:** 10.1002/alz.095238

**Published:** 2025-01-09

**Authors:** Kei Onn Lai, Nevin Tham, Lauren H Fairley, Roshan Naik, Yulan Wang, Sarah R Langley, Anna M. Barron

**Affiliations:** ^1^ Nanyang Technological University, Singapore Singapore; ^2^ Cardiff University, UK, Cardiff United Kingdom

## Abstract

**Background:**

The mitochondrial translocator protein (TSPO) is a biomarker of inflammation associated with aging and Alzheimer’s disease (AD). We have previously shown that TSPO plays a critical role in protective immune responses important in AD. Here we investigated the interaction between TSPO immunomodulatory function and aging in the hippocampus, a region severely affected in AD.

**Method:**

Using RNAseq, we investigated the role of TSPO in the aging hippocampal transcriptome. Multi‐weighted co‐expression network analysis (WGCNA) was used to determine the effect of TSPO deletion on aging and identify hub regulators of the molecular network in aging. We compared the TSPO‐dependent aging transcriptome signature with drug gene expression signatures in a perturbational signature library called Connectivity Map (CMap). NMR metabolomics was used to determine the effect of TSPO in the context of aging and inflammation on brain metabolites.

**Result:**

Aging resulted in reversal of inflammatory transcriptional signatures in TSPO‐KO hippocampus, with TSPO deletion drastically exacerbating inflammatory transcriptional responses in the aged whilst dampening inflammation in the young hippocampus. This TSPO‐aging interaction was linked to transcriptional control of interferon regulatory factors. Drugs such as heat shock protein inhibitors and topoisomerase inhibitors were identified to phenocopy the transcriptional signature characterizing the inflammatory response in TSPO‐dependent aging. Through NMR detection, we also noted the exclusive presence of methanediol, another form of formaldehyde, in the aged TSPOKO mice.

**Conclusion:**

The effect of TSPO in inflammatory responses is age‐dependent, an interaction which we linked to transcriptional control of interferon regulatory factors. This TSPO‐aging interaction is an important consideration in interpretation TSPO‐targeted biomarker and therapeutic studies, as well as *in vitro* studies which cannot model the aging brain.

